# Gene therapy for rare diseases – the case for acute intermittent Porphyria

**DOI:** 10.1186/1878-5085-5-S1-A143

**Published:** 2014-02-11

**Authors:** Konstantina Grosios

**Affiliations:** 1 uniQure B.V., Meibergdreef 61, Amsterdam 1105 BA, The Netherlands

## 

It could be argued that gene therapy is the ultimate personalised medicine and potentially preventive medicine too. There is little closer to the personalisation of medicine than the ability to repair or restore the function of a person’s own faulty genes, the core principle of gene therapy. With the first approval of gene therapy products in both the western and eastern world, this is increasingly close to becoming a reality in clinical practice. At the same time, the advances in scientific understanding and technological ability to analyse the human genome imply that gene therapy could also be used to prevent the development of disease.

Many rare diseases are monogenic diseases and as such are ideal candidates for gene therapy. One example is Acute Intermittent Porphyria (AIP). The disease is caused by mutations in the *porphobilinogen deaminase* (*pbgd*) gene which encodes one of the enzymes involved in the haem biosynthesis pathway. This gives rise to enzyme deficiency and in turn accumulation of intermediate toxic metabolites in the heam pathway. The disease which has prevalence in Europe of approximately 5.9 per million population, manifests as severe abdominal pain, mental symptoms and peripheral neuropathy leading to poor life quality. Current therapy targets the symptoms of the disease without being able to modify the pathology, let alone provide a cure. AMT-021 is an adeno-associate virus 5 (AAV5)-based gene therapy product that aims to specifically deliver and express PBGD in the liver cells of affected patients therefore restoring the defect in haem biosynthesis and avoiding the accumulation of associated toxic metabolites. Following a series of preclinical development programme and pivotal toxicology and safety studies AMT-021 entered clinical trials (Phase I) in December 2012. This was the first time a potentially curative treatment was tested in AIP patients and from a scientific perspective this was the first time an AAV-5 based gene therapy product was administered in humans. The trial is expected to be completed in the second half of 2014.

Outside the significance of this drug development programme for the treatment of patients with AIP and gene therapy for rare diseases, it also represents a successful example of an academic-industrial partnership that has been made possible by funding from the European Commission’s Framework Programme 7. The AIPGENE consortium right from the beginning aimed to harness excellent pre-clinical research and by following a very pragmatic and translational approach focused it on developing a much needed therapy for a rare disease while extending the scientific frontiers of gene therapy as well.

